# Exploring Links Between Industrialization, Urbanization, and Chinese Inflammatory Bowel Disease

**DOI:** 10.3389/fmed.2021.757025

**Published:** 2021-10-28

**Authors:** Guanglin Cui, Hanzhe Liu, Gang Xu, Jann-Birger Laugsand, Zhigang Pang

**Affiliations:** ^1^Research Group of Gastrointestinal Diseases, The Second Affiliated Hospital of Zhengzhou University, Zhengzhou, China; ^2^Faculty of Health Science, Nord University, Levanger, Norway; ^3^Faculty of Dental Medicine, Wuhan University, Wuhan, China; ^4^Department of Gastroenterology, South Campus of the First Affiliated Hospital of Zhengzhou University, Zhengzhou, China

**Keywords:** inflammatory bowel disease, industrialization, urbanization, environmental factors, incidence

## Abstract

**Background:** Evidence is emerging that the incidence of inflammatory bowel diseases (IBD) is dramatically increased in China, but with a geographic variation.

**Objectives:** We performed a review to summarize the link of accelerated industrialization, urbanization to changing trends in the incidence of IBD over the last three decades.

**Methods:** An electronic database search was performed in PubMed, Medline, EMBASE and Google Scholar (for English literature) and the China Science Periodical Database in Wanfang Data (for Chinese literature) from January 1990 to June 2020.

**Results:** By systematically analyzing the changing trends of gross domestic product (GDP) or GDP per capita, population migration from rural areas to cities and increasing incidence of IBD in parallel in different Chinese regions, an association between accelerated industrialization and urbanization and rising rate of IBD was shown. In which, rates of IBD incidence were higher in provinces with a high value of GDP per capita than those provinces with a low value of GDP per capita. Analysis of available epidemiological data revealed that the incidence of IBD was rising in parallel with increasing trends of both gross products of industry and urban population in Yunnan Province in a 14-year interval. Further evidence suggested that industrialization- and urbanization-induced subsequent changes in environmental factors, e.g., Westernized dietary habits and obesity, and work-related stress, might contribute to the increased risk of IBD in China. In addition, the preliminary results showed that urbanization and Westernized dietary habits might induce significant changes in gut microbiota profile that are possibly to increase the risk for IBD in Chinese.

**Conclusions:** Existing evidence to suggest that accelerated industrialization/urbanization is associated with the increasing incidence of IBD in China, which provides novel insights to study the possible mechanisms for the recent increasing incidence of IBD in newly industrialized and urbanized developing countries. In the future, the interaction between relevant environmental factors e.g., air/water pollution and IBD susceptibility genes in Chinese should be examined.

## Introduction

Inflammatory bowel disease (IBD) is a group of idiopathic and relapsing chronic inflammatory conditions of the gastrointestinal tract that are mainly represented by ulcerative colitis (UC) and Crohn's disease (CD) (1). Initially, Asia has been classified as an agricultural region with a low incidence of IBD for a long period (2, 3). However, compelling evidence from Ng et al. and other researchers has demonstrated a dramatic increase in the incidence of IBD in traditionally low-incidence regions across Asia (2, 4–11), where industrialization and urbanization have progressively been accelerated over the last three decades (12, 13). Historically, China was a traditional rural-agricultural social model country and over 90% of the land's population lived in rural areas for thousands of years. However, China has been undergoing rapid industrialization in the past 30 years, which is reflected in significantly increased gross domestic product (GDP) or GDP per capita. Together with urban-rural integration policies (14), fast industrialization may result in a high demand for factory labor and a considerable population migration from rural areas to cities (15). Studies showed that both industrialization and urbanization may take a toll on many aspects of the environment, i.e., crowding, increased air pollution and industrial waste, and work-related stress associated with poverty and unemployment (16). All these changes may contribute to the increasing risk for IBD in developing countries (10, 17). Because of its population size and rapid industrialization and urbanization speed, China represents an ideal model to study the relationship between industrialization/urbanization and IBD in developing countries. Previous studies have documented significant geographical variations in inflammatory bowel diseases (IBD) rates in China (10). The objectives of this review are to analyze the role of industrialization/urbanization, and subsequent environmental changes in geographical variation in IBD incidences in China and to discuss the potential role of these factors in contributing to the increasing risk for human IBD.

## Methods

### Search Strategy

For English literature, a search was performed in the PubMed, Medline, EMBASE and Google Scholar computerized bibliographic databases, and for Chinese literature, a search was performed in the China Science Periodical Database in the Wanfang database based on relevant search terms “industrialization,” “urbanization,” “environmental factor,” “gut microbiota,” “Westernized diet,” “air pollution,” “inflammatory bowel diseases,” “ulcerative colitis,” “Crohn's disease” and “China” both in English and Chinese, respectively, up to September 2020. After screening the abstracts, the articles deemed relevant were cross-referenced for additional manuscripts. In addition, statistical data on the changes in Chinese industry value and urbanization (population and rate) over time from the World Bank database (14, 18), Yunnan's gross industry product values between 2000 and 2010 from the Yunnan Provincial Bureau of Statistics (19), and the world ranking of GDP per capita from 2010 to 2015 from World Economic Net (20) were obtained.

## Current Results and Evidence

### Summary of Literature Selection

The initial search strategy identified 1,768 references (1,587 in Chinese and 181 in English). Finally, twenty one articles (10 in Chinese and 11 in English, see Table 1) with full text met the inclusion criteria after screening and eligibility selection for literature evaluation. In addition, statistical data on the changes in Chinese industry value and urbanization (population and rate) over time from the World Bank database (14, 18), Yunnan's gross industry product values and urban population changes between 2000 and 2010 from the Yunnan Provincial Bureau of Statistics (19), and the world ranking of GDP per capita from 2010 to 2015 from World Economic Net (20) were obtained.

**Table 1:** Included full-text publications and databases for this review.

**Author/references**	**Dates**	**Publishing languages**		**Patient size**		**Study design**	**Parameters**
		**Chinese**	**English**	**CD**	**UC**		
Zhu et al. (21)	1990–2000	+			101	Case-control	Environmental risk factors
Yuan et al. (22)	1982–2001	+			196	Case-control	Diet risk factors
Wang et al. (23)	2000–2002	+			43	Case-control	Environmental risk factors
Wang (24)	2002–2004	+		41		Case-control	Environmental risk factors
Jiang et al. (25)	2004		+		177	Case-control	Environmental risk factors
Li et al. (26)	2003–2006	+		12	128	Case-control	Environmental risk factors
Shi et al. (27)	2002–2005	+		51		Case-control	Environmental risk factors
Chinese-IBD-Work-Group (28)	2004–2005	+			745	Case-control	Environmental risk factors
Chen and Wang (29)	2006–2009	+			100	Case-control	Environmental risk factors
Ng et al. (3)	2011–2013		+	IBD 284 (Han ethic Chinese)		Case-control	Environmental risk factors
Miao and Miao (30)	1998–2013	+		194	3,225	Population-based	Incidence, environmental and relapse factors
Wang et al. (31)	2007–2010		+		1,308	Case-control	Environmental risk factors
Hsu et al. (32)	1995–2014		+	34/57,611	52/57,611	Retrospective matched-cohort study	Alcohol abuse
Niu et al. (33)	1998–2007		+	102	678	Case-control	Environmental risk factors
Cui et al. (34)	2013–2018	+		41		Case-control	Environmental risk factors
Ng et al. (35)	2011–2013		+	IBD 418 (from China mainland)		Prospective population-based	Urbanization and IBD incidence
Chen et al. (36)	2011–2012		+	26	46		Gut microbiota
Winglee et al. (37)	Unidentified		+	Rural 26,563	Urban 7,000,000	Population-based	Urbanization and Gut microbiota profile change
Chen et al. (36)).	2010		+	26	46		Gut microbiota
Ma et al. (38)	2014–2016		+	15	14		Gut microbiota
Zhou et al. (39)	2012–2013		+	72	51		Gut microbiota
*Databases* World-Economic-Net (20)	2000–2015	+					World ranking of GDP per capita
Yunnan Provincial Bureau of Statistics (19)	2000–2010	+					Statistical data of Chinese urbanization
Worldbank (18)	1990–2019		+				Statistical data of Chinese industry
Worldbank (14)	1990–2019		+				Statistical data of Chinese urbanization

### Evidence for the Increasing IBD Incidence During the Accelerated Industrialization Period in China

**Figure 1:**
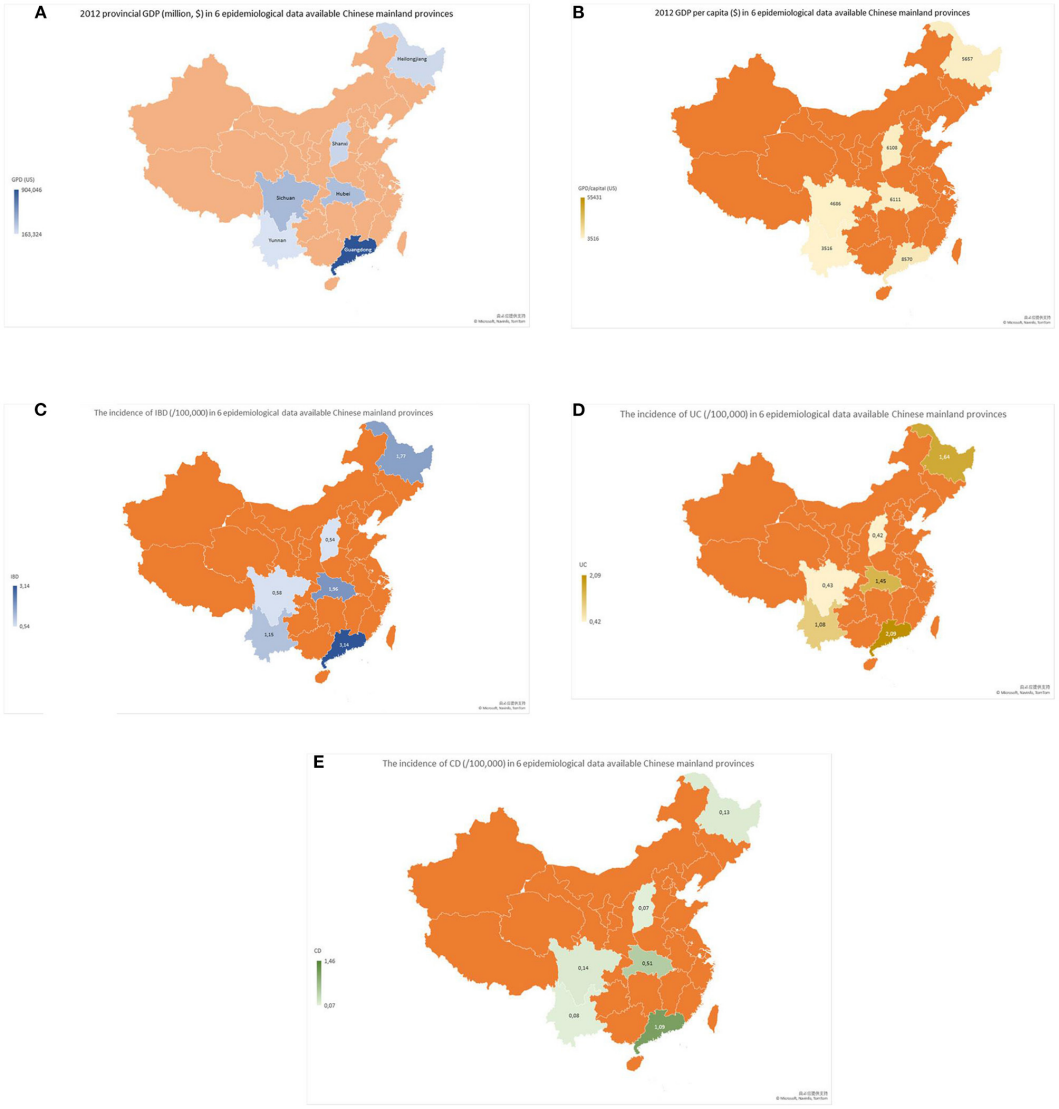
Maps of GDP (million $), GDP per capita ($), and IBD, UC and CD incidences (/1,000,000) in six Chinese mainland provinces with different levels of industrialization and available epidemiological data. The maps showed that provincial GDP **(A)** and GDP per capita **(B)** in Guangdong Province (southern China) were obviously higher than those in Sichuan and Yunnan Provinces (southwestern China). Interestingly, incidences of IBD **(C)**, UC **(D)** and CD **(E)** were also higher in Guangdong Province than in Sichuan and Yunnan Provinces. In addition, incidences of IBD **(C)**, UC **(D)** and CD **(E)** in Hubei Province (central China) and Heilongjiang Province (northern China) with a middle level of GDP per capita developed industrialization were higher than those in Sichuan and Yunnan Provinces (both in southwestern China).

**Figure 2:**
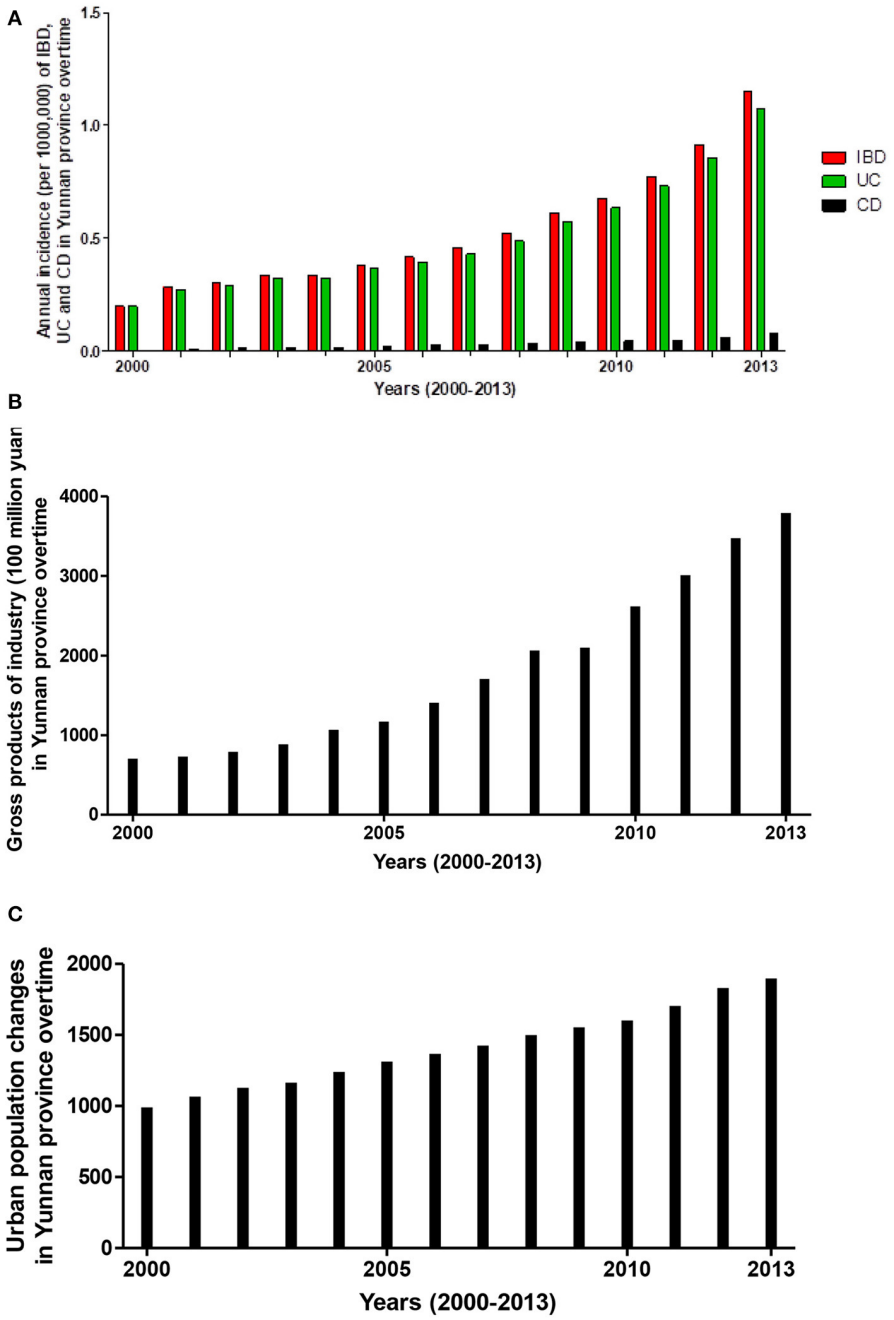
Increasing incidence trends of IBD, UC and CD showed a similar trend of gross products of industry in Yunnan Province over a 14-year interval (2000–2013). Statistical data showed that increasing incidences (/1,000,000) of IBD, UC and CD **(A)** paralleled the increasing gross products of industry [excluding construction **(B)**] and urban population [data source from the Yunnan Provincial Bureau of Statistics **(C)**] in Yunnan Province over a 14-year interval (2000–2013).

To analyze the impact of industrialization on IBD incidence, Ng et al. have previously used GDP as an index of industrialization level and found that rising GDP was closely associated with increasing IBD incidence in Asia-Pacific regions (3). Data from World Bank (18) showed that China has experienced a considerable industrial growth over the last 30 years, the increasing GDP value mainly come from industrialization. In this review, we were able to illustrate differences in IBD incidences between regions with higher and lower GDP or GDP per capita levels in epidemiological data available Chinese provinces (Figure 1). Data showed that Guangdong Province (located in southern China) with higher levels of GDP and GDP per capita levels (40), had higher incidences of IBD, UC and CD than Sichuan and Yunnan Provinces (both located in Southwest China), which had lower levels of GDP or GDP per capita (8, 30) (see Figures 1A–E). In addition, incidences of IBD in Heilongjiang (northern China) (41) and Hubei (Central China) Provinces (both traditional industrialized regions with a higher GDP per capita level) (25) were also higher than those in those two provinces located in Southwest China (8, 30) (see Figures 1B–D). From the maps (Figures 1A–C), we found that the value of the GDP per capita is better than the GDP to represent the industrialization level and consistent with the changing trend of IBD in the epidemiological data available Chinese regions. For example, Sichuan Province had a higher GDP value but lower IBD incidence than Hubei Province; this might mislead to a conclusion that a higher GDP value was associated with a lower IBD rate. However, a positive relationship between GDP per capita and IBD incidence (Figure 1C) was found when we used GDP per capita (Figure 1B) as an index to compare the difference in IBD incidences between Sichuan and Hubei. Finally, the increasing trends of incidences of IBD, UC and CD (Figure 2A) and gross products of industry (excluding construction) (Figure 2B) (19) in Yunnan Province (a province with traditionally low incidence) were shown in a very similar pattern in a 14-year (2000–2013) interval (30). Similar trends were also reported in Chinese populations in Taiwan, the incidence of IBD was gradually increased (42, 43), following the GDP per capita rise since the 1980s (20). These findings indicate that incidences of IBD, UC and CD in the regions with high rates of industrialization and urbanization are higher than those regions with low rates in China.

### Evidence for Fast Urbanization in the Increasing IBD Incidence in China

Recent epidemiological studies reported that population density and urbanization were associated with increasing rates of IBD in Asian regions with traditional low incidence (7, 35, 44). For example, Hong Kong is a highly urbanized Chinese city with a growing number of inhabitants, and the incidence of IBD in Hong Kong significantly increased from 0.10 in 1985 to 3.12/100 000 in 2014 (45). In China mainland, statistical data showed that the roughly urban population was increased from 300.17 million in 1990 to 848.43 million at the end of 2019 (14), and the rate of urbanization increased from 26.44% in 1990 to 60.31% at the end of 2019 (14, 46). With the accelerated rate of urbanization, China is now entering an acceleration incidence stage of IBD (13). Epidemiological evidence from Yunnan Province demonstrated that incidences of IBD were gradually increased from 0.198 (0.196 for UC, 0.002 for CD; refer to Figure 2A) in 2000 to 1.152 (7.035 for UC, 0.418 for CD respectively; refer to Figure 2A) per 100,000 inhabitants in 2013 (30). In the same period, the urban population of Yunnan province was increased from 9.9 million in 2000 to 18.9 million in 2013 (refer to Figure 2C). Rising rates of IBD (including both CD and UC) incidence (Figure 2A) were clearly in parallel with increasing trends of urban population (Figure 2C) in Yunnan Province in a 14-year interval (2000–2013). Among 3419 cases of IBD from 1998 to 2013, the percentages of IBD patients in urban residents were significantly higher than those in rural residents (small city vs. big city vs. rural: 33.87 vs. 48.58 vs. 17.55%; *P* < 0.01) (30). The geographic study suggested that the degree of urbanization is higher in southern and eastern Chinese regions than in southwestern and northern Chinese regions (47). Interestingly, IBD epidemiological data revealed an IBD incidence gradient from South to North and West China (35, 40, 41). For instance, the incidences of IBD (2.05 for UC and 1.09/100,000 for CD) in Zhongshan city, Guangdong Province (Southern China) (40) were much higher than that (1.64 for UC and 0.13/100 000 for CD) in Daqing City, Heilongjiang Province (Northern China) (41), and (0.43 for UC and 0.14/100 000 for CD) in Chengdu city, Sichuan Province (South-western China) (8). Therefore, current evidence from China suggests that regional variation in the incidence of IBD is likely associated with the different level of urbanization.

### Relevant Environmental Contributing Factors for Increasing Incidences of IBD in Chinese Populations

Fast industrialization and urbanization in China will inevitably cause a series of changes in the environment, by which increase the risk for IBD.

#### Westernized Lifestyle and Dietary Habits Are Progressively Being Adopted, and the Rate of Overweight/Obesity Is Increased

Chinese epidemiological studies have revealed that many diet factors in Chinese food may contribute to the increasing incidence of Chinese IBD (3, 10, 25, 30, 35, 48, 49), these factors have been summarized in our previous publication and readers could refer to it in detail (10). Current section will focus on the role of Westernized lifestyle and overweight/obesity in Chinese IBD. As we have known that rapid industrialization, urbanization, and economic and income growth might result in a dramatic shift from a traditional Chinese lifestyle progressively to a westernization lifestyle in urban inhabitants, which reflects in the increasing consumption of Westernized food and a high rate of overweight/obesity (50, 51). Which has been recognized as an inflammatory condition and increases the risk for future IBD, particularly for CD (52–54).

Evidences from Western lands supported that obesity was a risk factor for IBD and the incidence of IBD was rising in parallel with overweight/obesity in the Nurses' Health Studies (54, 55). Several studies have suggested that rapid growing rates of overweight/obesity in both rural and urban Chinese populations (56–58) could be, at least in partially, explained by the increased consumption of Westernized food (50, 59–62). Furthermore, a study showed that consuming more Westernized or meat diets might significantly increase rates of overweight/obesity and central adiposity in Chinese urban students (50). To date, although no studies have directly investigated the relationship between overweight/obesity and Chinese IBD incidence. However, many epidemiological studies have revealed that obesity risk factors might increase the risk for IBD (10, 55).

Increasing alcohol abuse is a worldwide health problem in modern industrial society. Aiming to assess the possible role of alcohol in IBD, Wang et al. (31) evaluated the association between alcohol consumption and IBD. They found that either light or heavy alcohol consumption could significantly increase the risk of developing UC in the Chinese population [light drinkers: odds ratios (OR) = 1.264, 95% confidence interval (CI): 1.073–1.490, *P* = 0.005; heavy drinkers: OR = 1.453, 95% CI: 1.122–1.882, *P* = 0.005] (31). Hsu et al. also reported that the alcohol intoxication cohort (*N* = 57,611) had a higher incidence of CD [adjusted hazard ratio (HR): 4.40, 95% CI: 2.58–7.51] and UC (HR: 2.33, 95% CI: 1.39–3.90) than the non-alcohol intoxication cohort (*N* = 230,444) in Chinese individuals in Taiwan during the 10-year follow-up (32). However, inconsistent results from Western nations were also reported. Porter et al. (63) reported that moderate alcohol consumption was associated with lower UC risk (adjusted HR: 0.35, 95% CI: 0.19–0.64) in the USA Millennium Cohort Study. Bergmann et al. reported that no relationship was found between alcohol consumption and the risk of UC and CD in six European countries (64). Therefore, further epidemiological studies with a large sample size are needed.

#### Changes in Gut Microbiota Profile

Similar to findings in Western CD patients (65, 66), increasing studies also focus on the role of gut microorganisms as possible causes of Chinese IBD. Chen et al. (36) reported an impaired composition of gut microbiota in both Chinese UC and CD patients based on the characterization of the fecal-associated microbiota and mucosa-associated microbiota, in which several butyrate-producing bacteria were significantly decreased, however *Escherichia-Shigella* and *Enterococcus* were increased. Ma et al. (38) found that altered composition of gut *Bacteroidetes* may have a negative impact on the development of Chinese UC. Zhou et al. (39) revealed that altered composition of microbiota has a predicative value for the diagnosis and evaluation of therapeutic response to infliximab treatment in Chinese patients with UC. Changes in gut microbiota profile in Chinese IBD were summarized in Table 2.

**Table 2:** Changed gut microbiota profile in Chinese IBD.

**References**	**Gut microbiota profile changes**	
	**UC**	**CD**
Chen et al. (36)	Butyrate-producing bacteria such as the genera Roseburia, Coprococcus, and Ruminococcus ↓, Escherichia-Shigella and Enterococcus ↑	Similar to UC
Ma et al. (38)	Proteobacteria ↑ the Escherichia genus ↑	Proteobacteria ↑↑ the Escherichia genus ↑ Bacteroidetes ↓
Zhou et al. (39)	Actinobacteria ↑ and Proteobacteria (Enterobacteriaceae) ↓ Firmicutes (Clostridiales) were correlated with disease severity. A significant increase in Clostridiales predicts an response to infliximab	Similar to UC

↑ *increased*;

↓ *decreased*.

More importantly, the composition and function of gut microbiota are regulated by numerous environmental factors, such as diet, overweight/obesity, intestinal inflammation, antibiotic use, and urbanization (67). Human studies found that a Westernized diet, with high animal protein and fats and low carbohydrates, might result in a changed composition of gut microbiota, in which *Bacteroides* was increased and *Prevotella* decreased (68). Furthermore, Winglee et al. (37) studied the compositional changes of the gut microbiome induced by urbanization in Hunan Province (South-Central region of China) and identified significant differences in the microbiota and microbiota-related plasma metabolites between urban and rural populations. Their data indicated that Chinese urban inhabitants had a changed gut microbiota profile, similar to that seen in the American population (37). Zhou et al. have recently revealed that gut microbiota profile in Chinese IBD patients was shown in a similar picture as Western IBD patients (39). These findings suggested a possible link between the changed gut microbiota profile induced by urbanization and the development of IBD in Chinese population (refer to Table 3).

**Table 3:** Changes in gut microbiota profile in relative with Westernized diet and urbanization in Chinese.

**Factors (references)**	**Gut microbiota profile changes in Chinese**
Westernized diet (68).	*Bacteroides* ↑; *Prevotella* ↓
Urbanization (37)	Profile becomes Westernized

↑ *increased*;

↓ *decreased*.

#### Work-Related Stress

The procession of industrialization and urbanization will increase work-related stress in traditional rural areas (69). Chinese epidemiological studies conducted in Yunnan Province revealed an apparent relationship between work-related stress and UC risk (30, 33), data showed that people with work-related stress had a higher overall risk (OR 1.732, 95% CI 1.142–2.628) for UC than those without (OR 1.235, 95% CI 0.749–1.743, *P* < 0.05) (33). However, this conclusion is not confirmed by the European study (70). Differences in culture and anti-stress ability between Eastern and Western populations may partially explain such inconsistent findings. Comparison studies showed that depression was much less common in Chinese populations than in American populations. Major depressive disorders in American populations have a nearly 20% incidence, but it is only 2% in Chinese populations (71, 72).

**Figure 3:**
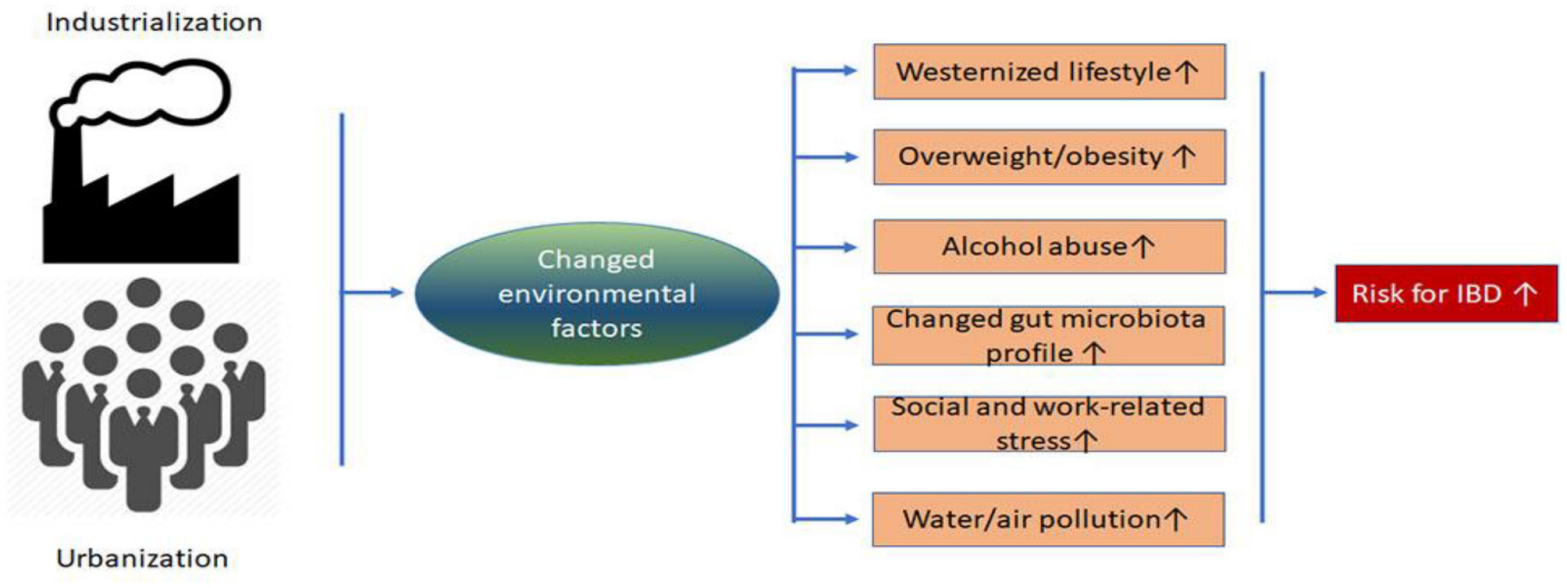
Schematic summarized environmental risk factors induced by industrialization and urbanization for IBD. Emerging evidence suggested that accelerated industrialization and urbanization in China increase the risk for IBD through relevant environmental factors.

The potential role of industrialization/urbanization and subsequent surrounding environmental changes as IBD risk factors is summarized in Table 4 and Figure 3. Other relevant environmental changes emerged after industrialization and urbanization (73–75), such as water and air pollution have been related to the development of IBD in other countries (76–79). However, to the best of our knowledge, no reports have examined the association between air/water pollution and IBD incidence in Chinese populations.

**Table 4:** Industrialization, urbanization, and relevant environmental risk factors identified in Chinese IBD.

**Risk factors**	**UC**	**CD**
Industrialization	+ (3, 40, 41)	+ (3, 40, 41)
Urbanization	+ (35, 40, 41)	+ (35, 40, 41)
Crowded living conditions		+ (27)
Westernized lifestyle		
*Dietary habit*		
Heavy sugar consumption	± (21, 22, 31)	+ (27)
Meat consumption	− (30, 33)	± (30)
Fried food intake	+ (22, 31, 33)	
Salty food intake	+ (33)	
Alcohol abuse	+ (31, 32)	+ (32)
*Work-related stress*	+ (30, 33)	
Changed gut microbiota profile	+ (36, 38, 39)	+ (36, 38, 39)
Water/air pollution	Studies no available in Chinese IBD	Studies no available in Chinese IBD

*+: Positive association with IBD; ±: both positive and negative association with IBD were reported*.

### Perspective of Changing Environmental Risk Factors in IBD

Understanding the impact of relevant environmental risk factors is crucial for formulating effective preventing and treating strategies for IBD.

#### To Prevent the Development and Release of IBD

Removal and modification of certain risk factors may reduce the triggering of IBD development and disease relapse in patients with IBD. For example, the gut microbiota contains mainly organisms from the *Bacteroides* and *Firmicutes phyla*, which can be regulated by several dietary components (67). This fact raises the attractive possibility that manipulating the composition of the gut microbiota could prevent overweight/obesity and then reduce the risk for IBD in humans.

#### To Improve the Clinical Outcomes of IBD

Westernized diet and urbanization may induce dysbiosis of gut microbiota (80, 81). Therefore, modifying the gut microbiota profile through diet, probiotics and fecal microbiota transplantation, etc., may reduce disease activity, disease course and hospitalization and finally improve clinical outcomes.

## Discussion

This review highlights the potential role of industrialization, urbanization, and relevant environmental risk factors in increasing IBD incidence in China and discuss the possible contributing factors on dramatically increasing incidence of IBD in newly industrialized and urbanized developing countries. By analyzing GDP or GDP per capita or populations in IBD epidemiological data available provinces, we were able to show different rates of IBD incidences in geographic regions with different level of industrialization and urbanization.

One of the key findings of current systematic analysis in this review is that incidences of IBD, UC and CD in the regions with high rates of industrialization and urbanization were higher than those regions with low rates. In the past 30 years, the rapid growth of Chinese GDP value mainly comes from the process of industrialization. Our analysis showed that the value of GDP per capita was better than GDP in order to be consistent with the changing trend of IBD in Chinese regions and might be a better index for industrialization because it excluded the influence of population added to provincial GDP. For example, the GDP of Sichuan Province was $377,819,000 and higher than that ($352,478,000) of Hubei Province in 2012. However, the value of GDP per capita in 2012 for Sichuan was $4,686/person and was lower than the value ($6,111/person) for Hubei Province. This was most likely because Sichuan Province had a larger population than Hubei Province. Analysis also showed that the IBD incidence in Shanxi (a province located in Central China) and with GDP per capita at a level of 6,108 US dollar was at a level of 0.54/100 000 (0.42 for UC and 0.07/100 000 for CD), which was much lower than that (IBD incidence 1.77, 1.64 for UC and 0.13/100 000 for CD) in Heilongjiang (a province located in Northern China). However, the value of GDP per capita in Shanxi province was slightly higher than that in Heilongjiang province (Shanxi vs. Heilongjiang: 6,108 vs. 5,657 US dollar). To explore the possible reasons for this inconsistency of IBD incidence between two provinces, we have further analyzed the composition of GDP for Shanxi and Heilongjiang provinces. We found that unlike provinces in Heilongjiang where GDP growth predominately comes from industries such as steel, oil and machine manufacturing that require a large number of workers. However, Shanxi is a province with abundant underground coal resources, it's GDP growth heavily relied on energy (coal) industry over the last three decades because the price of coal was gradually rising overtimes. Shanxi's coal production accounted 28% of Chinese coal production, which made Shanxi's coal industry as the biggest coal industry in China in 2000 (82). Due to highly automation in coal mining, most people in Shanxi were not involved in the coal industry. Therefore, the impact of industrialization on the people of Shanxi was not as big as it was on the people of Heilongjiang province, although the two provinces have a GDP per capita of about the same level. This phenomenon remains us that the differences in the GDP composition and variations in individuals should be considered, when GDP or GDP per capita is used as an solo index for industrialization level. In addition, the development of IBD is influenced by multiple factors. Shanxi is located in central China and is a region with profound Chinese traditional culture. In Shanxi, the traditional Chinese food habits are more preserved, which are reflected in more cereals, fibers, and vegetables, but less sugary foods and meats. Such diet components have been considered as protective factors for Chinese IBD (10, 25, 48). Furthermore, epidemiological studies confirmed a geographic variation in Chinese IBD incidences between southern and southwestern Chinese regions (10, 30), where different rates of industrialization and urbanization were observed (83). Finally, the incidence of IBD was increased with time and industrialization. Our analysis showed that the changing trend of IBD, UC and CD incidences in a 14-year (from 2000 to 2013) follow-up study was consistent with the step-up tendency of gross industry products in Yunnan Province (30). These analyses provided further supportive evidence for the hypothesis that industrialization is associated with the development of IBD.

Ongoing expanding industrialization and China's urban-rural integration policies might result in accelerated urban population recruitment (15). Studies have shown that fast accelerated industrialization and urbanization in China may lead to a remarkable change in the dietary pattern between urban and rural regions in the past three decades, and Westernized food has become popular in urban areas (50). A Westernized diet high in fat and carbohydrates and low in fiber is implicated in the increasing incidence of IBD (81). Traditionally, Chinese daily food intake contains high cereals and legumes and many vegetables and fibers but less fat, meat, and milk. However, current Chinese diet recipes have been skewed in a Westernized style (60). Such food intake and nutrient change could induce overweight/obesity in both Chinese children and adults (50, 60). In contrast to the report from Chinese children, a systematic analysis from the United States showed that higher rates of overweight and obesity were often found among American children living in rural areas than children in urban areas (84). Such inconsistent conclusions might reflect the difference in dietary composition and economic level between Chinese and American rural families. The economic level of Chinese families was generally lower than that of American families living in rural areas. In addition, many rural Chinese families still retain more traditional diet habits and consume less fat, red meat and milk but eat more vegetables, fruits, and cereals than urban families today.

The gut microbiota profile alteration has been demonstrated in both Chinese and Western patients with IBD. Scientific evidence has strongly indicated that environmental factors such as diet can modulate the composition and function of gut microbiota and intestinal homeostasis (85), which is associated with the onset and development of IBD. Interestingly, a study reported that compositional patterns of abundant microbes in the urban Chinese population become Westernized and similar to those in the urban American population, which was hypothesized to be induced by urbanization (37). Furthermore, researchers found that Italian children who consumed a typical Western diet had a much less diverse microbiome than African children who consumed mainly legumes, grains and vegetables (86). These findings supported the hypothesis that the gut microbiota profile can be regulated by environmental factors and have opened a new opportunity for a therapeutic strategy through modulating the gut microbiota profiles by diet, probiotics, and fecal microbiota transplantation to improve the clinical outcomes in patients with IBD.

Finally, two studies reported that work-related stress increased the risk for Chinese UC (30, 33), which needs further confirmation.

Limitations and challenges should be addressed and discussed. First, China is a big country with hugged population. The level of industrialization and urbanization varies cross the country. To better interpret the influence of urbanization on IBD incidence, it is better to make a figure to show the IBD gradient along with urbanization/migration across the country. However, current epidemiological studies of Chinese IBD incidence are not nationwide population-based studies and only available in several provinces. Therefore, we were unable to analyze the gradient changes of IBD incidence along with urbanization that covers most provinces of China. Second, the role of industrial risk factors such as wastewater and air pollution have not been evaluated in Chinese IBD populations. Therefore, the most urgent task is to conduct a continuous nationwide epidemiological survey as soon as possible, which might address these concerns.

## Concluding Remarks and Recommendations for Public Health Authorities and Professionals

When summarizing the existing clinical epidemiological data in this review, a strong body of evidence suggested that the dramatically increasing incidence of IBD in traditionally low-incidence Chinese regions can be, at least in partially, explained by industrialization, urbanization and subsequent changed environmental factors (see Figure 3). However, current Chinese epidemiological studies are not nationwide population-based studies and are limited by small sample sizes and lack of robust longitudinal follow-up. Therefore, the most urgent task is to conduct a continuous nationwide epidemiological survey as soon as possible. Furthermore, to explore the reasons for geographical variation in IBD in Chinese regions with different industrialized and urbanized levels, future efforts should be focused on examining the effects of other environmental changes induced by industrialization, such as water and air pollution, on IBD susceptibility genes. Finally, since the composition and function of gut microbiota can be regulated by environmental factors, the effect of modifying the gut microbiota profile through diet and probiotics in newly industrialized and urbanized regions should be considered.

## Author Contributions

All authors listed have made a substantial, direct and intellectual contribution to the work, and approved it for publication.

## Conflict of Interest

The authors declare that the research was conducted in the absence of any commercial or financial relationships that could be construed as a potential conflict of interest.

## Publisher's Note

All claims expressed in this article are solely those of the authors and do not necessarily represent those of their affiliated organizations, or those of the publisher, the editors and the reviewers. Any product that may be evaluated in this article, or claim that may be made by its manufacturer, is not guaranteed or endorsed by the publisher.

## Abbreviations

CD: Crohn's disease
CI: confidence interval
GDP: gross domestic product
HR: hazard ratio
IBD: inflammatory bowel diseases
OR: odds ratios
UC: ulcerative colitis.
